# Continuous or interrupted pledgeted suture technique in stented bioprosthetic aortic valve replacement: a comparison of in-hospital outcomes

**DOI:** 10.1186/s13019-024-02754-3

**Published:** 2024-04-04

**Authors:** Bardia Arabkhani, Sebastien Gonthier, Veronica Lorenz, Samuel Deschamps, Jama Jahanyar, Marin Boute, David Vancraeynest, Stefano Mastrobuoni, Gebrine El Khoury, Laurent de Kerchove

**Affiliations:** 1 Department of Cardiovascular and Thoracic surgery, UC Louvain Saint Luc, Brussels, Belgium; 2 Department of Cardiovascular Diseases, Cliniques Universitaires St. Luc, and IREC/CARD UCLouvain, Brussels, B-1200 Belgium; 3 Department of Cardio-Thoracic Surgery, Erasmus Medical Center (EMC), Dr. Molewaterplein 40, Rotterdam, 3015 GD The Netherlands; 4 Division of Cardiothoracic Surgery, Department of Surgery, Queen’s Heart Institute, John A. Burns School of Medicine, University of Hawaii, Honolulu, HI USA

**Keywords:** Aortic valve replacement (AVR), Suture technique, Continuous technique, Interrupted-pledgeted technique, Valvular hemodynamic performance

## Abstract

**Background:**

There is ambiguity in the literature regarding the continuous suture technique (CST) for aortic valve replacement (AVR). At our center, there has been a gradual shift towards CST over the interrupted pledgeted technique (IPT). This study aims at comparing outcomes for both techniques.

**Methods:**

We performed a retrospective analysis of a single-center study of patients undergoing AVR between January 2011 and July 2020. Patients were divided into two groups: Continuous suture technique and interrupted pledget-reinforced sutures. The pre-operative and In-hospital clinical characteristics and echocardiographic hemodynamics (i.e. transvalvular gradients and paravalvular leakage) were compared between CST and IPT.

**Results:**

We compared 791 patients with CST to 568 patients with IPT (median age: 73 and 74 years, respectively, *p* = 0.02). In CST there were 35% concomitant procedure vs. 31% in IPT (*p* = 0.16). Early mortality was 3.2% in CST versus 4.8% in IPT (*p* = 0.15), and a second cross-clamp due to a paravalvular-leak in 0.5% vs. 1.2%, respectively (*p* = 0.22). The CST was not associated with new-onset conduction-blocks mandating pacemaker implants(OR 1.07, 95% CI 0.54–2.14; *P* = 0.85). The postoperative gradients on echocardiography were lower in CST compared to IPT, especially in smaller annuli (peak gradients: 15.7mmHg vs. 20.5mmHg, in valve size < 23 mm, *p* < 0.001).

**Conclusions:**

The continuous suture technique was associated with lower postoperative gradients and shorter cross-clamp time compared to interrupted pledgeted technique. Differences in paravalvular leaks were non-significant, although slightly less in the continuous suture technique. There were no further differences in valve-related complications. Hence, continues suture technique is safe, with better hemodynamics compared to the interrupted pledgeted technique. This may be of clinical importance, especially in smaller size annular size.

**Supplementary Information:**

The online version contains supplementary material available at 10.1186/s13019-024-02754-3.

## Introduction

Surgical aortic valve replacement (SAVR) has been the gold standard for the treatment of severe aortic valve disease. In the last decade however, transcatheter aortic valve implantation (TAVI) has become increasingly important in the treatment of aortic stenosis [1]. Nevertheless, according to the most recent ESC/EACTS guidelines for the management of valvular heart disease, SAVR is still recommended in younger patients who are at low risk for surgery or patients who are operable and unsuitable for TAVI [2]. Amongst biological prostheses available for SAVR, the stented bioprostheses are associated with very satisfactory long-term outcome and are the most commonly used types of prosthesis. However, in recent years the so-called “sutureless” bioprostheses have also been developed to reduce operative time and for utilization in less-invasive surgical approaches, with promising early results [3]. Although, comparable to TAVI, there is a higher rate of pacemaker implantation, compared to conventional bioprostheses [4].

Another issue is structural valve degeneration (SVD) of the biological prosthesis, which determines the durability of the valve. There are several factors that affect the rate of SVD, including anti-calcification treatments of the biological tissue, the configuration of the bioprosthesis patients’ age, potential prosthesis-patient mismatch, which is associated with transvalvular pressure gradients postoperatively [5]. Higher pressure gradients postoperatively are associated with early progressive SVD, probably due to turbulence through the bioprosthesis. Apart from the choice of the type of bioprosthesis, the only parameter that allows to optimize the durability of the prosthesis is therefore its size and possibly the way it is implanted in order to obtain the lowest pressure gradients at the end of the procedure [6].

Suturing techniques differ according to the surgeon’s preference and habits. The most common implantation technique is the pledget-reinforced mattress sutures. A less frequently used technique is the (semi)continuous suture, which is associated with shorter cross clamp- and cardiac bypass time [7].

However, there are studies that associates the continuous suture technique to higher rates of postoperative paravalvular leak and reoperation, compared to the pledget-reinforced sutures [8, 9]. Although these studies were performed with mechanical prosthetic valves, in our opinion these are not suitable for implantation with the continuous suture technique.

In order to identify the potential impact of suture techniques on biological stented prosthetic valve hemodynamics and operative outcomes, we reviewed and compared our patients who underwent AVR, utilizing the pledget-reinforced mattress versus the continuous suture technique.

## Patients and methods

### Patients

From January 2011 to June 2020, all consecutive patients, aged 18 years and older, who underwent aortic valve replacement (+/- concomitant procedures) with a stented bioprosthetic valve, either an Edwards Magna Ease or an Abbott Trifecta and Trifecta GT, were consecutively enrolled and entered in an electronic database and included in this study. Postoperative echocardiographic exam is routinely performed before discharge in all patients. In-hospital clinical outcome (i.e. mortality, pace-maker implants, cerebrovascular and other valve-related events) and echocardiographic outcome (i.e. paravalvular leakage, transvalvular gradients) were compared between the continuous suture and the single interrupted pledgeted sutures technique. In general, there was a gradual shift towards utilization of the Edwards Magna prosthesis during the years. The cohort consists of two groups:

1. Patients undergoing AVR with the continuous suture technique, CST.
2. Patients undergoing AVR with single interrupted pledgeted sutures, IPT.

During the last decade, there was a gradual shift towards the utilization of the CST, which was started as an innovative approach in AVR. This technique was adapted by most of surgeons in our center during the last few years.

To evaluate the effect of suture technique on gradients in different sizes of valve prosthesis, we compared the mean and peak transvalvular gradient postoperatively (predischarge transthoracic echocardiography) in three subgroups of patients with (1) small valve size, namely valve sizes 19 and 21; (2) most frequently used valve size 23; and (3) the relatively bigger valve sizes 25 or greater. Since the two types of bioprosthesis may have different hemodynamic characteristics, we compared the above mentioned valve sizes separately for patients receiving a Magna Ease and Trifecta prosthesis. An additional comparison was performed within patients who had a postoperative mean gradient of more than 15mmHg to association of the effect of suture technique with postoperative clinically meaningful higher gradient. Since the effective orifice area measurements were not available in all patients, we have compared the body surface area (BSA) in separate groups, as a surrogate measurement to ensure there were no differences between the CST and IPT groups.

This study was approved by the Review Ethics Board of Université_Catholique de Louvain, and patient informed consent was waived (StudyID: 2022/21MAR/132).

### Surgical technique

After median full or partial upper sternotomy, cardiopulmonary bypass (CBP) was initiated via central aortic and right atrial cannulation, and an aortic root vent was placed. Then the aorta was cross-clamped and blood cardioplegia was administered in antegrade fashion. In case of aortic valve insufficiency, cardioplegia was administered directly into the coronary ostia. A left ventricular vent was inserted via the right superior pulmonary vein. The field was flooded with CO2. The operation was performed in normothermia. After aortotomy, three 4–0 Ethibond retraction sutures were placed above the commissures and pulled and tightened to the skin, in a way to optimize exposure of the annulus. Hereafter, the aortic valve was removed, and the aortic annulus was thoroughly decalcified.

For the continuous suture technique (CST), three polypropylene 2.0 sutures were used, one for each sinus. In larger valves (27 mm or greater) occasionally four sutures were utilized. The suture was started at the level of the commissure between the non- and the left coronary sinus and moving clockwise, starting from the prosthetic valve sewing ring to the ventricular side of the annulus. Then the prosthetic valve was parachuted by pulling on the sutures at the level of the commissures. With a blunt nerve hook, the sutures were tightened and the suture line was evaluated from the inside by opening the leaflets. Then the knots were tied behind the commissures. For the interrupted pledgeted suture technique (IPT), 12 to 15 pledgeted 2.0 Ethibond sutures were passed from the ventricular side of the annulus through the aortic side of the prosthetic valves’ sewing ring. The aortotomy was closed with two continuous sutures of polypropylene 4.0. Finally, a systematic transesophageal echocardiographic evaluation was performed. Postoperative anticoagulation management was with Aspirin only unless there was new onset atrial fibrillation or other indications for oral anticoagulation. All patients underwent structural, predischarge transthoracic echocardiographic exam with measurements of transvalvular mean and peak pressure gradients, and evaluation for (para)valvular leak.

### Statistical analysis

Categorical data are presented as counts with proportions. Continuous data are presented as means (standard deviation; range) when normally distributed or medians (interquartile range) when not normally distributed. For categorical data, the chi-squared test or the Fisher’s exact test was used for comparison between groups, and for continuous data, an unpaired t-test or Mann-Whitney U test, depending on distribution. Univariable logistic regression analysis was performed to control the association between the surgical technique and outcomes (i.e. paravalvular leak, need for pacemaker implantation, and postoperative gradients higher than 15 mmHg) by possible confounders.

Candidate variables with a P-value of < 0.10 or clinically relevant were assessed in a multivariable model. Supplemental Table 1 displays the details.

All tests were performed 2-sided, and a P value of < 0.05 was considered statistically significant. For the statistical analysis R (version 4.2.0, available at: www.r-project.org) was used.

## Results

Median hospital stay was 8 (IQR 6) days, for both CST (791 patients, 58%) and IPT (565 patients, 42%). The Edwards Magna Ease prosthesis was used in 65% of patients and the Abbott Trifecta and Trifecta GT, available since 2016, was used in 35% of patients. Table 1. shows preoperative patient characteristics in both groups. The preoperative patient characteristics were comparable between the CST and IPT groups, except for age, which was slightly lower in the CST group (73- vs. 74 years, *p* = 0.02) and endocarditis as indication for operation, which was more frequent in the CST (2.9% vs. 0.6%, *p* = 0.001). Table 1 provides detailed information on preoperative characteristics. Table 2 displays preoperative echocardiographic characteristics. Cardiopulmonary bypass time for isolated AVR was 72 vs. 83 min, and cross-clamp time 51 vs. 58 min in CST and IPT, respectively. In-hospital mortality for combined procedures was 3.2% and 4.8% (*p* = 0.15) for CST and IPT, respectively, and 2.3% vs. 2.9% (*p* = 0.63) for isolated AVR. Suture technique was not associated with hospital mortality: OR 0.65 (95% CI 0.37–1.13, *p* = 0.22).

**Table 1:** Preoperative patient characteristics

	All patients (N = 1356)	CST (N = 791)	IPT (N = 565)	p-value
**Age (mean, sd/median, IQR)**	72.4 (8.8)	73 (67–80)	74 (66–78)	0.02
**Gender (Male)**	58%	56%	60%	0.07
**Diabetes Mellitus**	25%	26%	25%	0.90
**Hypertension**	75%	74%	76%	0.28
**Creatinine level (mg/dl)**	1.08 (0.86–2.34)	1.11 (0.87–2.62)	1.04 (0.85–1.45)	0.10
**Hypercholesterolemia**	66%	68%	65%	0.27
**NYHA**				0.53
I	24%	24%	24%	
II	45%	46%	44%	
III	28%	27%	28%	
IV	3%	3%	4%	
**Previous cardiac surgery**	12.1%	12.7%	11.3%	0.45
**BSA (median, IQR)**	1.9 (1.8–2.2)	2.0 (1.8–2.2)	1.9 (1.7–2.1)	0.10
**BMI (median, IQR)**	28 (26–33)	28 (25–32)	28 (24–33)	0.13
**Euroscore II (mean, sd)**	3.6 (4.3)	3.6 (4.5)	3.5 (4.1)	0.80
**Previuos CVA or carotid** **stenosis > 70%**	4.2%	2.2%	2.0%	0.41
**Concomittant procedures**				
CABG	33.5%	35.0%	31.3%	0.16
MVP	6.3%	7.3%	4.9%	0.07
MVR	5.4%	4.9%	6.2%	0.32
TVP	2.1%	2.1%	1.9%	0.79
Ascending aorta	7.1%	7.2%	6.9%	0.81
AF ablation	2.6%	2.5%	2.6%	0.90
Septal myectomy	4.5%	4.4%	4.6%	0.89
ASD closure	0.9%	0.9%	0.9%	0.99
VSD closure	0.1%	0.1%	0%	0.40
**Indication for operation**				0.16
Stenosis	87.6%	85.3%	89.4%	
Regurgitation	6.0%	6.6%	5.1%	
Mixed type	6.4%	8.1%	5.5%	
**Endocarditis (active)**	3.5%	2.9%	0.6%	0.001
**Full sternotomy**	88.5%	88.2%	88.9%	0.70
**Mini-sternotomy** **(Upper Hemi)**	11.5%	11.8%	11.1%	0.62

CST: Continues suture technique; IPT: Interrupted pledgeted technique; NYHA: New York Heart Association; BSA: Body surface area; BMI: Body mass index; CABG: Coronary artery bypass graft; NVP: Mitral valve plasty; MVR: Mitral valve replacement; AF: Atrial fibrillation; ASD: Atrial septal defect; VSD: Ventricular septal defect

**Table 2:** Preoperative echocardiographic characteristics

	All patients (N = 1356)	CST (791)	IPT (565)	P-value
**Peak gradient (mmHg) mean, sd**	74 (27)	73 (26)	76 (28)	0.06
**Mean gradient (mmHg)**	45 [17]	44 [17]	46 (18)	0.04
**Annular size (mm)**	24 [15]	23 (21–25)	24 (21–25)	0.59
**LVF**				0.87
Good (> 50%EF)	81.7%	81.9%	81.3%	
Moderate (30–50%)	14.8%	14.8%	14.8%	
Poor (< 30%)	3.5%	3.3%	3.9%	
**No. of Cusps**				0.001
Unicuspid	0.8%	1.3%	0.2%	
Bicuspid	23%	21.4%	25.4%	
Tricuspid	73.2%	75.4%	70.1%	
Prosthetic valve	2.9%	1.9%	4.4%	

CST: Continues suture technique; IPT: Interrupted pledgeted technique; LVF: Left ventricular function

### Comparison of PVL, need for pacemaker implants and stroke

A second cross-clamp was required due to paravalvular-leak on intra-op TEE: 0.5% in CST vs. 1.2% IPT (*p* = 0.22). Paravalvular-leak on TTE at discharge was 0.6% in CST vs. 1.3% in IPT (*p* = 0.23). The CST was not associated with paravalvular leak at discharge; also after adjustment for age, previous cardiac surgery and endocarditis (OR 0.51, 95% CI 0.16–1.60; *P* = 0.25).

There were 2.9% pacemaker implant in CST compared to 2.6% in IPT (*p* = 0.86) in all patients, and in isolated AVR, excluding patients with preoperative conduction disorders (i.e. second-degree or higher atrioventricular block or pacemaker rhythm; 19 patients in CST and 21 patients in IPT) this was 2.1% vs. 3.4% in CST and IPT (*p* = 0.45), respectively. In the multivariable logistic regression analysis, the suture technique was not associated with higher rates of new-onset conduction disorders mandating pacemaker implants, adjusted for concomitant procedures and endocarditis: OR 1.15, 95% CI 0.58–2.27 (*p* = 0.15).

Table 3 shows details on operative and postoperative outcome.

**Table 3:** Operative and postoperative event outcome

	All patients (*N* = 1356)			Isolated AVR (*N* = 621)		
	CST (*N* = 791)	IPT (*N* = 565)	P-value	CST (*N* = 347)	IPT (*N* = 274)	P-value
ECC minutes (median, IQR)	89 (48)	94 (53)	0.01	72 (23)	83 (28)	0.001
Cross-Clamp minutes (median, IQR)	66 (40)	69 (39)	0.08	51 [15]	58 [17]	0.001
Hospital mortality	3.2%	4.8%	0.13	1.7%	2.9%	0.39
PM implantation	2.9%	2.6%	0.86	2.1%	3.4%	0.45
Paravalvular leakage	0.5%	1.2%	0.22	0.6&	1.9%	0.25
Stroke	1.1%	1.7%	0.48	0.3%	1.3%	0.33

CST: Continues suture technique; IPT: Interrupted pledgeted technique; ECC: Extra corporal circulation; AVR: Aortic valve replacement

There were 1.1% postoperative, predischarge strokes (both transient ischemic and cerebrovascular accident) in CST compared to 1.7% in IPT (*p* = 0.48) in all patients, and 0.3% and 1.3%, in CST and IPT (*p* = 0.33) in isolated AVR, respectively.

### Comparison of postoperative hemodynamics (transvalvular gradients)

**Fig. 1:**
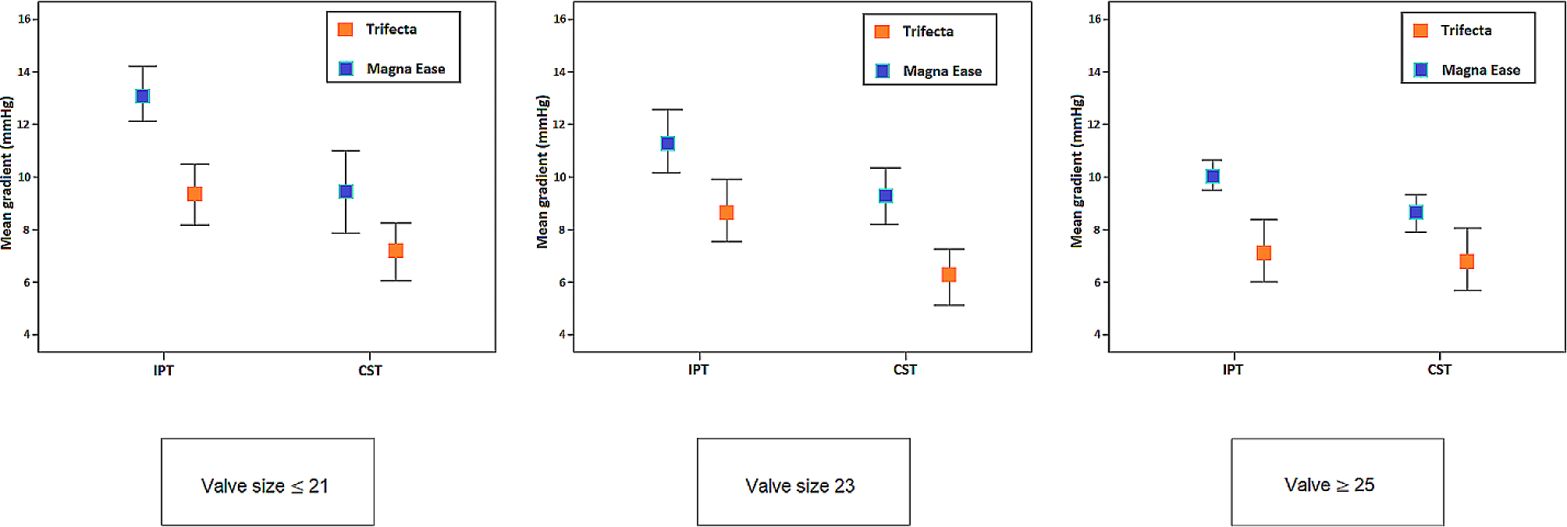
Predischarge transvalvular mean gradients for different valve sizes and type of prosthesis

The postoperative gradients on echocardiography were lower in CST compared to IPT, especially in smaller prosthesis. For prosthesis size 19 + 21, and size 23 the peak and mean transvalvular gradient were significantly lower in the CST group. A subgroup analysis of peak and mean transvalvular gradients of the Trifecta and Magna Ease valve separately, comparing CST to IPT within each type of valve prosthesis showed also lower gradients in the CST group, except for patients receiving a Trifecta valve size 25 or greater. In the latter there were no statistically significant differences. Table 4 shows details for different valve size and type of prosthesis. The BSA was comparable between CST and IPT, for the three subgroups. Supplemental Table 2 shows details about the postoperative gradients and BSA in subgroup of patients within different prosthesis sizes. Figure 1 shows postoperative, predischarge transvalvular mean gradients for different valve sizes, suture technique, and separate type of valve prosthesis.

Subgroup analysis showed more patients with postoperative mean gradients of > 15 mmHg in the IPT cohort than in CST (Supplemental Table 3).

**Table 4:** Postoperative gradients in subgroup of patients with different prosthesis sizes and type, for different suture technique

		N	19 + 21	p-value	N	23	p-value	N	> 23	p-value
**Peak gradient mmHg (sd)**										
Magna	IPT	43	24.0 (11.4)	0.02	117	21.3 (8.8)	0.001	165	18.3 (7.5)	0.01
	CST	36	18.3 (10.0)		142	18.4 (5.9)		364	16.1 (6.4)	
Trifecta	IPT	37	17.7 (6.7)	0.003	98	16.2 (7.3)	0.001	65	13.8 (7.1)	0.45
	CST	38	13.7 (6.5)		89	12.4 (5.0)		107	13.1 (5.5)	
**Mean gradient mmHg (sd)**										
Magna	IPT	43	13.2 (6.5)	0.01	117	11.5 (5.0)	0.001	165	10.0 (4.3)	0.001
	CST	36	9.8 (4.8)		142	9.6 (3.2)		364	8.6 (3.7)	
Trifecta	IPT	37	9.3 (3.6)	0.003	98	8.2 (4.0)	0.001	65	7.0 (4.3)	0.67
	CST	38	7.1 (3.6)		89	6.4 (2.9)		107	6.8 (3.1)	

CST: Continues suture technique; IPT: Interrupted pledgeted technique, N: Number of patients available for analysis; The numbers (19, 21, 23) indicate the size of prosthesis as indicated by the companies

## Discussion

This study shows that stented bioprosthetic aortic valve replacement, using the continuous suture technique, is safe and feasible. Continuous suture technique is associated with reduced transvalvular gradients, especially in smaller annular sizes, compared to the interrupted pledget-reinforced technique, for both the Magna Ease and the Trifecta prosthesis. Moreover, cardiopulmonary bypass and cross-clamp times are shorter in the continuous suture technique, and there is no difference in other valve-related outcomes.

Paravalvular leak after aortic valve replacement is an important issue, which, if untreated, could results in impaired hemodynamics [10]. The continuous suture technique has earlier been suggested to increase the risk of paravalvular leak after AVR compared to the pledget-reinforced sutures [8, 9], leading to more reoperations. However, these studies are performed in patients with mainly a mechanical prosthesis and a mixed cohort of mitral and aortic valve replacements. In our study, the continuous suture technique was not associated with increased rates of PVL. Nevertheless, we believe that application of the CST in mechanical valves, which have a flat shaped “annular” sewing ring, is not appropriate, as the shape of the ring forces placement of the sutures below the commissures into the muscular part anteriorly and fibrous part posteriorly, and at the level of the “basal ring”. This may potentially lead to a tear or rupture of the annulus there, especially at the commissure between left coronary and right coronary cusp, at the muscular part. Accordingly, this can lead to higher rates of PVL, or possibly pseudoaneurysms of mitro-aortic continuity. For this reason, we use the CST only for stented bioprostheses and not for mechanical valves.

Another issue is the need for pacemaker implants after aortic valve replacement, which is associated with higher hospital stay and mortality. This is true for both in SAVR and TAVI, which was elucidated from the PARTNER trial data by Nazif et al. [11]. In our study the CST was not associated with more pacemaker implants compared to the IPT. Nevertheless, there are several factors that may increase the risk of a new-onset conduction disorder, such as e.g. age, valve anatomy, endocarditis. Most patients eligible for SAVR are relatively young and active with a low risk profile and a long life expectancy. These patients can be particularly affected by the need for a pacemaker in term of quality of life and clinical outcomes. Hence surgeons should be cautious to prevent damage to the conduction system. The continuous suture technique does not initiate any additional risk with this regard and could be applied safely.

Furthermore, there is no consensus in the literature regarding hemodynamic performance of bioprosthetic valves in different suture techniques. Tabata et al. have suggested that using a pledgeted technique to implant a biological stented valve in aortic position is associated with higher prosthesis-patient mismatch, in small annular sizes [10]. However, Ugur and colleagues reported that in smaller annular sizes (implanted prosthesis of 19- and 21 mm), comparing pledgeted technique to non-pledgeted, there is no difference in outcomes with regard to prosthesis-patient mismatch, and they suggest that the differences between the findings may be due to the use of the Trifecta valve prosthesis, which may have better hemodynamic performance [12]. We have found lower transvalvular gradients in similar valve sizes in the CST compared to IPT. As one may suspect these gradients are higher in smaller sizes, and both the Magna Ease and the Trifecta showed the same differences, except for the bigger sizes (25 and bigger), where we did not find any difference only in the Trifecta valve. Especially in smaller annuli, where a prothesis size 19 or 21 are used, this may be of clinical importance, although other characteristics may also be important for these differences in gradient such as left ventricular outflow tract (septum) obstruction. Since the prosthesis size labeling is rather arbitrary and does not represent the actual annular size, we could not compare the size of the specific type of prosthesis to one another.

Due to lack of postoperative echocardiographic effective orifice area (EOA) data, we were not able to identify the PPM that is suggested by Pibarot et al. and widely accepted [13], with the transvalvular pressure gradient as a major factor. However, we have identified the BSA which is used in a calculation to determine indexed EOA and subsequently PPM, and the BSA was comparable between the CST and IPT cohort. Nevertheless, for comparable mean BSA and same valve size and valve brand representation, we found more patients with higher mean and peak gradient in the IPT compared to the CST, which suggests that the higher gradients are presumably due to the pledgets used in the IPT cohort, leading to some obstruction of the LVOT. Moreover, suture technique may affect the hemodynamic outcome of AVR with bioprosthetic stented prosthesis. In patients with a small aortic annulus, the IPT may reduce annular diameter by 1 mm or more [14]. Additionally, in an experimental study Capelli and colleagues evaluated the overall hydrodynamic performance under identical conditions in pledgeted and non-pledgeted sutured biological stented prosthetic valves, and computational fluid dynamics analyses were performed, which showed flow disturbances in pledget-armed sutures, which in turn increased the mean pressure gradient and decreased the effective orifice area [15].

One can argue to use a stentless valve in small annular size, but this is usually extending the operation to an aortic root replacement, which may not be necessary in the majority of patients. Besides, the hemodynamic performance of the third-generation supra-annular stented bioprosthesis is generally similar to that of stentless valves [16]. Nevertheless, long-term follow-up of hemodynamic performance and analysis of degeneration of the bioprostheses over time is warranted to evaluate the effects of suture technique on the hemodynamics. Another option in smaller valves could be a sutureless or rapid deployment bioprosthetic valve. However, these valves may induce higher atrioventricular blocks compared to conventional bioprosthetic valves. Additionally, sutureless valves do not allow for relative oversizing or root augmentation. More important, since sutureless valves are relatively new and there are no comprehensive studies with long term follow-up in low risk and young patients, we believe that sutureless valve are not the most appropriate choice in these patients.

Furthermore, the in-hospital mortality and risk of a stroke were low and comparable between the CST and the IPT, as one may expect. There were no major differences in preoperative characteristics between the two techniques used for AVR. We can assume that the suture technique is not associated with higher hazard of major valve-related hospital events.

Finally, a potential issue may be a presumed higher risk of infectious endocarditis due to the pledget (Teflon) material that is used in the IPT. Although there are no comprehensive data available in the literature, the incidence of a prosthetic valve endocarditis is suggested to be 6 per 1000 cases with a cumulative risk of around 6% after 10 years in high-risk patients [17]. A recently published study by Velders et al. [18] clinical outcomes were comparable between patients undergoing AVR with pledgeted and non-pledgeted sutures, up to 5 years of follow-up with comparable endocarditis rates. Nevertheless, pledget use was associated with a slightly smaller EOA eventually. Notably, only 15% of patients in the non-pledget cohort were treated with the continuous suture technique.

In the current study, the follow-up is too short to evaluate the risk of endocarditis. However, we do hypothesize that there may be a slightly higher risk when using pledgets, especially in immune incompetent patients. Long-term follow-up may provide further insights in the future. We are more cautious in using the CST in patients with extended annular calcification and endocarditis, especially when not accustomed in using this technique.

## Limitations

A limitation is that this study contains only in-hospital outcomes. Although we do not expect the valve-related events to be different at long-term follow-up, the echocardiographic parameters (i.e. transvalvular gradients and paravalvular leakage) may change during follow-up and affect patient outcome. Another issue is the use of two type of prosthesis, Edwards’ Perimount Magna and Abbott’s Trifecta. The valves have distinct characteristics. However, the difference in transvalvular gradients was observed also in subgroups of the different valves, over different annular sizes in both suture techniques. Furthermore, the number of events are low for the examined outcome, which makes analyzing the data challenging in term of statistical testing, however, the main goal of this study was to show the safety and efficacy of the CST, which could be addressed adequately.

In conclusion, the continuous suture technique is safe to implement for bioprosthetic aortic valve replacement and is associated with lower transvalvular gradients. Moreover, there is no additional risk of paravalvular leak or newly onset conduction disorders. Hence, the continuous suture technique should be considered a valuable technique for aortic valve replacement with stented biological prosthesis, especially in smaller annular sizes. Long-term follow-up of hemodynamic performance and analysis of degeneration of the bioprostheses over time is warranted to evaluate the effect of suture technique on the long-term hemodynamics.

### Electronic supplementary material

Below is the link to the electronic supplementary material.

Supplementary Material 1

## Data Availability

Data will be shared upon reasonable request to the corresponding author.

## Abbreviations

SAVR: Surgical Aortic Valve Replacement
TAVI: Transcatheter Aortic Valve Implantation
CST: Continuous Suture Technique
IPT: Interrupted Pledgeted Technique
SVD: Structural Valve Degeneration
EOA: Effective orifice area
BSA: Body surface area
