# Supplementary material for: Continuous or interrupted pledgeted suture technique in stented bioprosthetic aortic valve replacement: a comparison of in-hospital outcomes
Source: J Cardiothorac Surg. 2024 Apr 4;19:174. doi: 10.1186/s13019-024-02754-3 (PMC10996201; doi:10.1186/s13019-024-02754-3)
Supplement: Supplementary file 1 — Supplementary Material 1 [file 13019_2024_2754_MOESM1_ESM.docx]

**Supplemental material**

**Table S1.** Preoperative variables tested in an univariable logistic regression model (pacemaker implantation, paravalvular leakage, and mortality)

| **Variable** | **OR** | **95% CI** | **p-value** |
| --- | --- | --- | --- |
| **PM implantation** |  |  |  |
| Suture technique | 0.41 | 0.12-1.40 | 0.15 |
| Concomittant procedures | 1.14 | 0.33-3.90 | 0.84 |
| Endocarditis | 5.50 | 1.17-25.84 | **0.03** |
| Type of prosthesis | 1.33 | 0.42-4.22 | 0.63 |
| Previous cardiac surgery | 1.73 | 1.11-12.53 | **0.03** |
| BSA | 0.23 | 0.02-3.36 | 0.28 |
| Gender | 1.29 | 0.48-4.44 | 0.68 |
| Hypertension | 0.67 | 0.20-2.24 | 0.52 |
| Size of prosthesis | 0.44 | 0.13-1.48 | 0.19 |
| **Paravalvular leakgae** |  |  |  |
| Age | 0.98 | 0.92-1.04 | 0.41 |
| Gender (Male) | 3.72 | 0.81-18.02 | **0.09** |
| Suture technique (CST) | 0.50 | 0.16-1.59 | 0.24 |
| Hypertension | 0.67 | 0.20-2.25 | 0.52 |
| Previous cardiac surgery | 3.72 | 1.11-12.50 | **0.03** |
| BSA | 0.93 | 0.09-9.53 | 0.95 |
| Endocarditis | 5.49 | 1.17-25.78 | **0.03** |
| Type of prosthesis | 1.33 | 0.42-4.21 | 0.63 |
| Size of prosthesis | 0.42 | 0.05-3.33 | 0.41 |
| Surgeon | 1.00 | ------------ | 0.99 |
| **Mortality** |  |  |  |
| Age | 1.04 | 1.01-1.08 | **0.02** |
| Gender (Female) | 0.38 | 0.21-0.68 | **0.001** |
| Suture technique | 0.65 | 0.37-1.13 | 0.22 |
| Hypertension | 0.91 | 0.49-1.70 | 0.76 |
| Creat level | 1.0 | 0.99-1.0 | 0.22 |
| Hypercholesterolemia | 0.81 | 0.46-1.42 | 0.46 |
| NYHA  I  II  II | 0.85 1.98 2.50 | 0.38-1.89 0.92-4.24 0.66-9.49 | 0.67 0.10 0.18 |
| Previous cardiac surgery | 2.28 | 1.17-4.44 | **0.02** |
| BSA | 1.00 | 0.99-1.00 | 0.13 |
| Euroscore II | 1.10 | 1.06-1.14 | 0.001 |
| Previuos CVA or carotid stenosis >70% | 1.97 | 0.69-5.67 | 0.21 |
| Diabetes mellitus | 1.20 | 0.65-2.22 | 0.56 |
| Concomittant procedures | 2.05 | 1.17-3.57 | **0.01** |
| Endocarditis | 1.72 | 0.52-5.72 | 0.38 |
| Type of prosthesis | 1.50 | 0.86-2.62 | 0.18 |

Table S2. Postoperative gradients and BSA in subgroup of patients with different prosthesis sizes.

|  | **19-21 (N=190)** | |  | **23 (N=453)** | | |  | ≥25 (N=711) | |
| --- | --- | --- | --- | --- | --- | --- | --- | --- | --- |
|  | CST (N=87) | IPT (N=103) | P-value | CST (N=233) | IPT (N=1220) | P-value | CST (N=471) | IPT (N=242) | P-value |
| **Peak gradient (mmHg, sd)** | 15.7 (8) | 20.5 (9) | 0.000 | 16.1 (6) | 19.0 (9) | 0.001 | 15.4 (6) | 17.0 (8) | 0.01 |
| **Mean gradient (mmHg, sd)** | 8.2 (4) | 11.0 (5) | 0.001 | 8.4 (4) | 10.0 (5) | 0.001 | 8.1 (4) | 9.1 (4) | 0.02 |
| BSA m2 (mean+sd or median+IQR) | 1.70 (0.16) | 1.71 (0.19) | 0.72 | 1.83 (0.20) | 1.88 (0.20) | 0.30 | 1.99 (0.28) | 2.00 (0.22) | 0.76 |
| Magna, N (%) | 37 (43%) | 47 (46%) | 0.77 | 143 (61%) | 121 (55%) | 0.18 | 358 (76%) | 174 (72%) | 0.24 |
| Trifecta, N (%) | 50 (57%) | 56 (54%) |  | 90 (39%) | 99 (45%) |  | 113 (24%) | 68 (28%) |  |

CTS: Continues suture technique; IPT: Interrupted pledgeted technique; BSA: Body surface area

**Table S3.** Patients with postoperative mean gradient > 15 mmHg, per type of prosthesis and suture technique and for different valve sizes

|  |  |  |  |  |  |  |  |  |  |
| --- | --- | --- | --- | --- | --- | --- | --- | --- | --- |
|  | **19-21 (N=31/190; 16.3%)** | |  | **23 (N=45/453; 9.9%)** | | | **≥25 (N=59/711; 8.3%)** | | |
|  | CST | IPT | p-value | CST | IPT | p-value | CST | IPT | p-value |
| **Mean gradient** **> 15 mmHg (%)** | 9.2% | 22.3% | 0.02 | 3.4% | 16.8% | 0.00 | 5.3% | 14.1% | 0.00 |
| BSA m2 (mean+sd or median+IQR) | 1.74 (1.60-1.81) | 1.82 (1.73-1.92) | 0.13 | 1.75 (0.19) | 1.96 (0.20) | 0.01 | 2.03 (0.24) | 2.04 (0.25) | 0.32 |

CTS: Continues suture technique; IPT: Interrupted pledgeted technique; BSA: Body surface area
